# Leading medical laboratory professionals toward change readiness: a correlational study

**DOI:** 10.1093/labmed/lmad091

**Published:** 2023-10-03

**Authors:** Taryn L Waraksa-Deutsch

**Affiliations:** Division of Cytopathology, Fox Chase Cancer Center, Philadelphia, PA, US; Department of Health Science, Bay Path University, Longmeadow, MA, US

**Keywords:** laboratory medicine, leadership, change readiness, continuous improvement

## Abstract

**Background:**

To remain effective in the dynamic health care landscape, the laboratory must embrace the continuous improvement mindset to support a culture of change, and leadership must facilitate the change process, mitigating perceived barriers of change readiness in followers.

**Methods:**

This quantitative study was designed to determine whether there is an association between leadership style (Multifactor Leadership Questionnaire [MLQ]) and change readiness (3-component model [TCM] commitment to change/Employee Commitment Survey, and whether leadership style predicts change readiness. Laboratory professionals (n = 718) were recruited through national societies to complete a combined MLQ-TCM survey instrument. Multivariate analysis of variance, Pearson correlations, and multiple regression analyses were performed.

**Results:**

A significant correlation between leadership style and change readiness (transformational leadership [TL] and affective commitment to change, *r*(716) = .12, *P* = .002; passive-avoidant behavior and continuance commitment to change, *r*(716) = .25, *P* < .001) and between leadership style and leadership outcomes (TL and effectiveness, *r*(716) = .90, *P* < .001) was identified. Transformational leadership was a significant predictor of change readiness (β = .17, *P* < .05).

**Conclusion:**

It is recommended that laboratory leaders use transformational leadership or situational leadership to improve followers’ affective commitment to change and reduce followers’ continuance commitment to change, thus improving commitment to continuous improvement. Leaders should also limit passive-avoidant behavior.

## Introduction

At the forefront of laboratory research is innovation and technology, whereas the foci of laboratory medicine as a profession includes training, professional development, staffing shortages, and burnout prevention or response. However, leadership within the profession and its impact on process outcomes through continuous improvement has not been proficiently examined. In other occupations, leadership style and methods have been thoroughly examined for their impact on organizational outcomes.^1^ As demonstrated by the response to the COVID-19 pandemic, a significant construct of professional preparedness desired in laboratory professionals is change readiness. Change readiness is defined as a measure of confidence that a person, team, or organization is committed, capable, and culturally inclined to embrace a proposed project or initiative that would lead to the desired target of change.^2^

Continuous improvement, as a form of change, is necessary to remain effective and evolve in health care. Antony et al^3^ share 3 main barriers to successful continuous improvement projects: resistance to change, lack of leadership support and commitment, and incompetent teams; whereas Erlingsdottir et al^4^ suggest that leaders can enable effective continuous improvement implementation by coaching the process, promoting autonomy, and providing the necessary resources. Leadership should help employees understand that a culture of continuous improvement can help reduce errors and avoid personal blame, and by appreciating and adopting the continuous improvement mindset, employees are doing their part for themselves, their organization, and their patients.^5^ Leaders must identify and effectively use appropriate change management tools to reduce employee resistance to change and promote readiness and adaptability, yet it is not clear whether leadership styles or methods are associated with change readiness in laboratory medicine.

### Purpose of the Study

This correlational study was designed to determine whether there is an association between leadership style as a change management tool and change readiness via commitment to change (CTC) in laboratory medicine, affecting laboratory professionals as both leaders and followers. There are limited data available regarding medical laboratory professionals’ perceptions of change and whether resistance and readiness are related to leadership style, communication issues, lack of resources and commitment, or a combination of various factors.^6^ Although studies have demonstrated an association between transformational leadership (TL) and continuous improvement initiatives through harnessing CTC across multiple sectors, there is no evidence that this relationship exists in laboratory medicine.

### Hypotheses

Two hypotheses were made for this study. Hypothesis 1 (H1) is that there is a significant association between leadership style (defined by the MLQ) and change readiness (measured by the 3-component model [TCM] of CTC/Employee Commitment Survey). Hypothesis 2 (H2) is that leadership style (defined by the MLQ) is a significant predictor of change readiness (measured by the TCM).

### Change Readiness via CTC

Weiner’s theory of organizational readiness for change posits that 2 collective affective states are related to change readiness: change efficacy and CTC.^7^ Herscovitch and Meyer define CTC as an individual’s mindset that binds them to the pathway or course of action an organization takes to achieve an improvement or change initiative.^8^ The TCM of CTC consists of 3 levels (affective, continuance, and normative commitment to change).^9^ Bouckenooghe et al^9^ opine that CTC behavior is driven by beliefs, such as inherent benefits or intrinsic motivation to remain with the organization throughout the change process (affective commitment to change, ACC), the risk analysis or perception of costs associated with change (continuance commitment to change, CCC), and a moral obligation or duty to support the change (normative commitment to change, NCC). ACC is most often correlated with positive work performance, mentorship, and reduced burnout; CCC is stress-inducing and depletes energy, leading to employee burnout and turnover; and NCC is influenced by externally-driven CCC and internally-motivated ACC, with higher levels of NCC correlated with increased stress and emotional exhaustion.^9-11^

### Full Range of Leadership

Bass’ full range leadership model conceptualizes that all leaders exhibit five leadership styles to some extent: laissez-faire leadership (LF), management by exception-passive (MBE-P), management by exception-active (MBE-A), contingent reward (CR), and the four Is of TL. The optimal leadership profile, as posited by Bass and Riggio,^12^ features LF as the least-used style and TL as the most frequently displayed style. Passive-avoidant behavior (PAB) is defined by nonaction or passivity where leaders remove themselves from their responsibilities (LF) or only act after a complaint is received (MBE-P), contributing to a blame culture.^1,12,13^ Lutz Allen et al^14^ determined that LF is negatively associated with building a psychological climate for both change readiness and organizational creativity due to a lack of leadership communication and unwillingness to facilitate an environment of transformational change. Although MBE-P is considered a form of transactional leadership according to Bass and Riggio,^12^ the style falls under PAB within leadership measurement instruments for its inaction or passivity.

Transactional leadership focuses on contractual relationships, structured management, formal delegation, and reinforcement systems that revolve around rewards and penalties.^12,15^ Perceptions of risk and safety drive commitment to organizational goals, enabling success preventative MBE-A leadership.^12^ MBE is a corrective transaction and it is therefore deemed less effective than CR and TL due to a lack of motivation involved.^12,16,17^ CR motivates employees by rewarding behavior with social and economic incentives, but transactional leadership may not be able to sustain CTC in long-term continuous improvement initiatives as well as TL due to changes in reward attractiveness.^12,18^

As the theoretical framework driving this study, TL is well recognized and accepted as an effective leadership strategy across the world.^12^ TL is known for its adaptability and empowerment, inspiring a shared vision to engage followers in their personal and professional growth while collectively working toward the organization’s mission and strategic initiatives.^1^ Ledlow and Stephens posit that the most effective transformational leaders in health care use transactional and TL simultaneously as an adaptive style to address the unique needs of their followers and the ongoing changes within the health care system.^1^ The effectiveness of TL is based on the impact the leader has on the follower across 4 psychological mechanisms or competencies: idealized influence (II), individualized consideration (IC), inspirational motivation (IM), and intellectual stimulation (IS).^12^

## Methods

### Participants, Sampling, and Data Collection

During an 8-week period in October and November of 2022, a survey was distributed online via SurveyMonkey to the American Society for Clinical Pathology (ASCP) and the American Society of Cytopathology (ASC) online listservs. All currently employed or retired medical laboratory professionals and pathologists, including supervisors, managers, and midlevel directors, within the United States were eligible for this study due to their relative experience working under some form of higher leadership.^12^ All medical laboratory professionals aged 18 years or older of any gender, educational background, length of experience, certification status, laboratory discipline, and setting were eligible to participate. International laboratory professionals who are proficient in written English language and work within the United States were also included in the study to better understand a potential association between leadership style and change readiness within ever-diversifying US health care organizations regardless of ethnicity, race, or nationality and to close the literature gap regarding laboratory medicine leadership in the United States. Membership in professional societies was not a requirement, but sampling procedures actively recruited members of the ASCP and ASC. Medical laboratory students and pathology residents without previous or current employment within a laboratory were excluded. Medical laboratory professionals who do not work in the United States were excluded due to the study’s intended understanding of leadership and change readiness in United States health care. Laboratory professionals who are not proficient in the written English language were excluded from the study, as the survey and related study documents were only offered in English. Although unanticipated, all professional or laboratory leaders who did not report to a higher level of leadership were excluded because the survey instrument used in this study was intended to measure an association between leadership style and subordinate change readiness.

### Instrumentation

Two previously validated measures, the MLQ 5X Short and the TCM Employee Commitment Survey, were combined in a 74-item survey and distributed online via SurveyMonkey to measure leadership style and change readiness (see Supplemental Materials). Following the consent statement and demographic questions, the combined MLQ-TCM survey presented statements to which the participant responded based on their degree of frequency or level of agreement/disagreement on a 5-point Likert scale.

### Multifactor Leadership Questionnaire

The MLQ 5X Short measures scales of TL, transactional leadership, and PAB in the form of 45-items, with 36 items on leadership style and 9 items on leadership outcomes.^12^ The MLQ Rater Form asks subordinates to rate a leader’s behavior. As a 9-subscale questionnaire, the MLQ examines 4 subscales associated with the 4 psychologic mechanisms of TL: IC, II (II-attributed [IIA] and II-behavior [IIB]), IM, and IS; 2 facets of transactional leadership: CR and MBE-A; and 2 subscales of PAB or nonleadership styles: MBE-P and LF. The ninth subscale measured the leadership outcomes of efficiency (EFF), extra effort (EE), and satisfaction (SAT). The MLQ has been psychometrically validated across various sectors, making it 1 of the most frequently used leadership questionnaires in research for its internal reliability, convergent validity, and discriminant validity.^12,19,20^ The MLQ does not have to remain a stand-alone measure, as the instrument was previously paired with the Implementation Leadership Scale, demonstrating acceptable internal consistency across all subscales, an excellent fit via confirmatory factor analysis, and convergent validity via moderate to high correlations between the MLQ and Implementation Leadership Scale.^19^

### TCM of Employee Commitment Survey

The TCM of commitment was originally established by Meyer and Allen in 1991 and later revised in 2004 as an 18-item Employee Commitment Survey with 6 items assessing each of the 3 well-validated subscales or levels of commitment.^21^ The TCM survey can be used to measure other foci of commitment, such as CTC, without negatively affecting reliability or validity, which spurred a CTC extension of the TCM.^8^ This extension of the revised survey was used in this project to measure an employee’s CTC within their organization on 3 levels: ACC, NCC, and CCC. Herscovitch and Meyer^8^ determined that the CTC extension of the TCM yielded alpha coefficients of .94, .94, and .86 for ACC, CCC, and NCC, respectively, which is greater than the original TCM with an average internal reliability of .82, .73, and .76 for affective, continuance, and normative commitment to the organization, respectively.

### Data Analysis

Data was analyzed via SPSS version 29 for Windows 11. Cronbach’s alpha coefficient was performed to determine the internal reliability of the combined MLQ-TCM.^8^ The MLQ-TCM responses were scored according to item, subscale, and scale, yielding 9 overarching results for each participant: 3 leadership scores (TL, transactional leadership, and PAB), 3 leadership outcomes (EFF, EE, and SAT), and 3 CTC scores (ACC, CCC, and NCC).^12,21^ Response frequencies and percentages for each survey item and the mean and standard deviation for each item and item subscale for the overall study sample were calculated. A 1-way multivariate analysis of variance (MANOVA) was also performed to assess the differences of demographic categorical data on change readiness via CTC scales.

A Pearson product-moment correlation coefficient (Pearson’s *r*) was used to assess the strength and direction of potential associations between the scale and subscale averages of both survey elements.^22^ The parametric correlation was used to determine the strength and direction between each of the 3 leadership styles (transformational, transactional, and PAB) as independent variables and each of the 3 types of CTC (affective, normative, and continuance) as dependent variables. Because an association was identified between leadership style and change readiness via CTC, a multiple regression analysis was performed to determine which leadership style, if any, is a significant antecedent or predictor of change readiness.^22,23^ Analyzing 2-tailed hypotheses, an alpha level of .05 (*P ≤* .05) was deemed statistically significant.^22^

### Ethical Approval

This study was approved by a private university’s institutional review board on September 23, 2022 (IRB # 2022_Waraksa).

## Results

### Demographic Characteristics of the Study Sample

**Figure 1.**
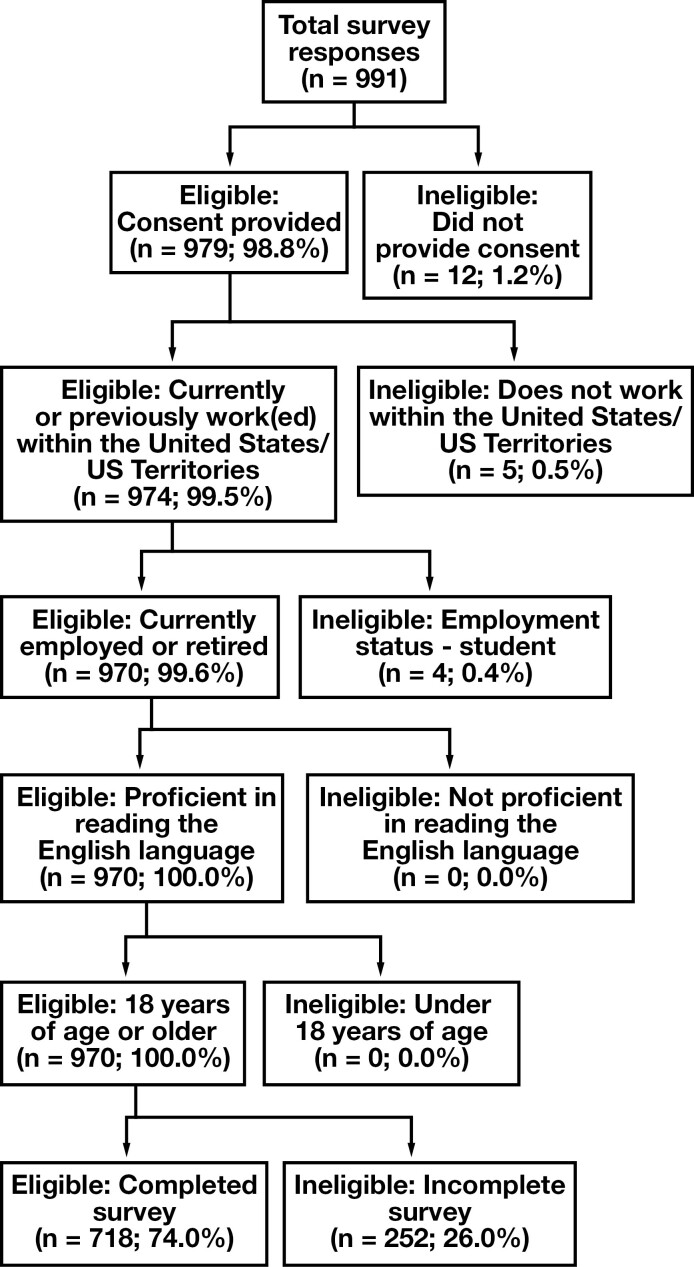
CONSORT flow diagram.

On survey closure, 991 individual responses were recorded. A CONSORT diagram was used to depict the flow of participant eligibility (FIGURE 1). Twelve recruited participants (1.2%) did not provide consent to participate in the research study and were therefore excluded. Of the 979 laboratory professionals (98.8%) who consented to the study, 5 participants (0.5%) reported that they did not work within or retire from a laboratory within the United States or United States Territories and were excluded. Of those 974 participants (99.5%) who work within or have retired from the United States, 4 participants (0.4%) reported that they are students and were deemed ineligible to participate in the study. A total of 970 eligible participants (99.6%) reported that they are currently employed or retired from a laboratory within the United States, proficient in reading the English language, and 18 years of age or older. Of the 970 eligible participants, 252 laboratory professionals (26.0%) did not respond to any of the MLQ-TCM instrument questions beyond the demographic questions and were consequently excluded from analysis due to an incomplete survey response. Therefore, 718 participants with completed survey responses were included in the analysis as the final study sample size.

**Figure 2.**
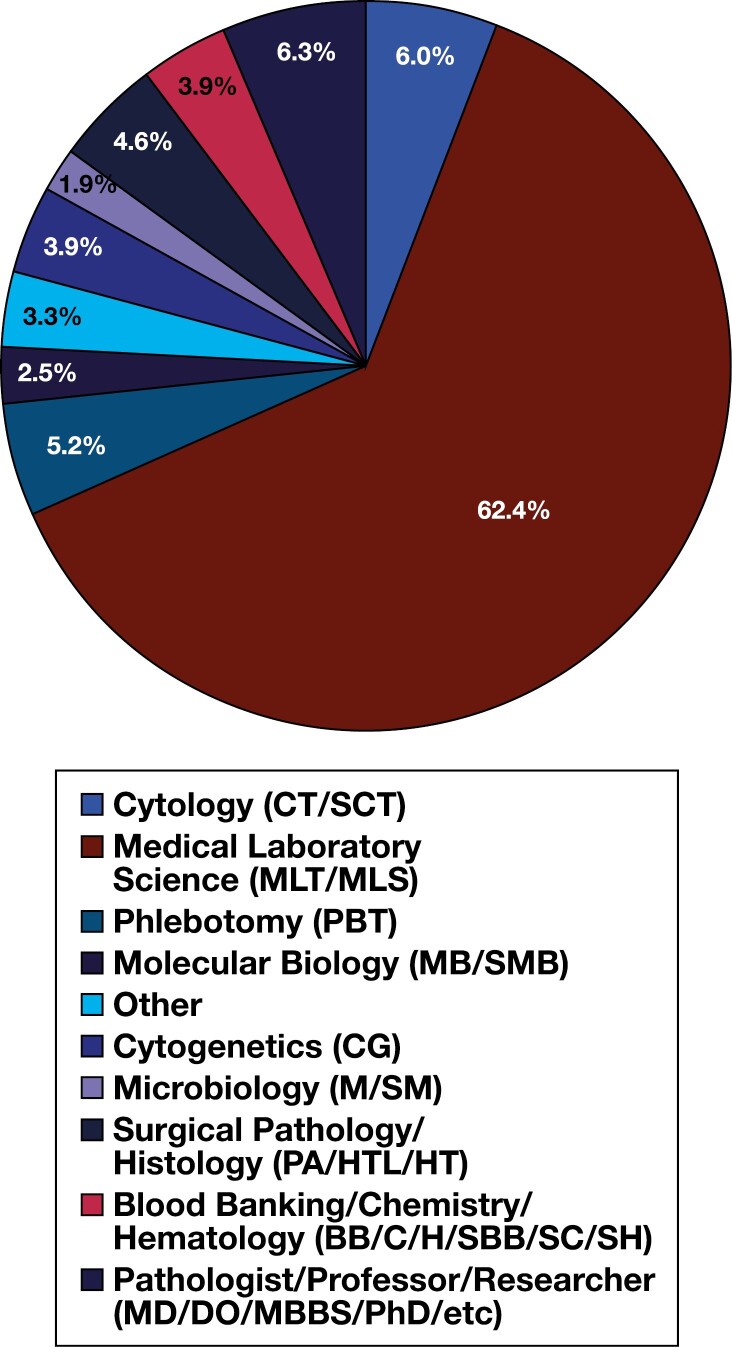
Sample laboratory disciplines/ASCP certifications. Note: “Other” laboratory disciplines include Flow Cytometry (SCYM); administration, such as those certified as Diplomats in Laboratory Management (DLM); generalists; and those certified in multiple disciplines.

**Figure 3.**
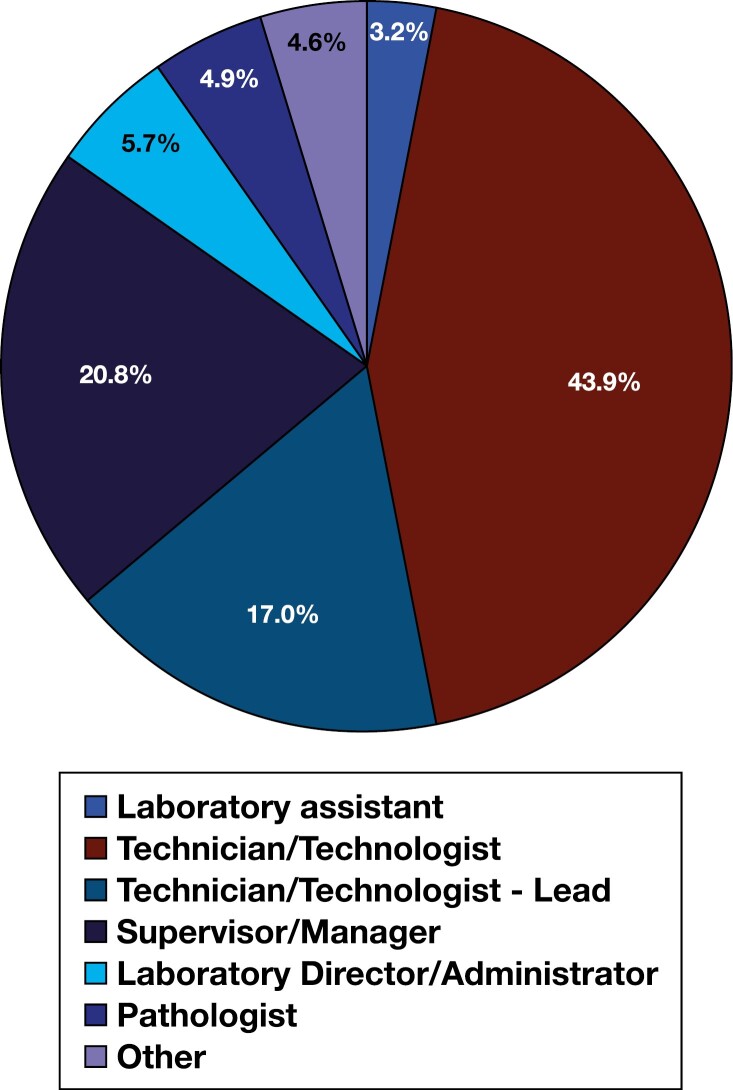
Sample reported laboratory roles. Note: “Other” roles include pathologist assistant, LIS analyst, quality specialist, educator/professor, and consultant.

Detailed participant demographic characteristics can be found within TABLE 1. Of the 718 study participants, most of the sample identified as Caucasian (White) (75.9%), female (78.4%), and between the ages of 30 and 39 (34.0%) or 40 and 49 (25.6%). As for education and work experience, most of the study participants reported that they work full-time (87.5%), have earned either a bachelor’s degree (56.4%) or a master’s degree (21.4%), have at least 10 years of experience in the laboratory (61.5%), and work in a hospital-based laboratory (77.0%). Additionally, the majority of the participants reported a laboratory discipline of medical laboratory science (MLT/MLS) (62.4%) and a role of technician/technologist (43.9%), lead technician/technologist (17%), or supervisor/manager (20.8%) (FIGURES 2 and 3, respectively).

**TABLE 1.** Demographic characteristics of survey sample

Characteristic	No. (%)	Characteristic	No. (%)
Gender		Laboratory discipline	
Female	563 (78.4)	Cytology (CT/SCT)	43 (6.0)
Male	144 (20.1)	Medical Laboratory Science (MLT/MLS)	448 (62.4)
Nonbinary	2 (0.3)	Phlebotomy (PBT)	37 (5.2)
Prefer not to answer	9 (1.3)	Molecular Biology (MB/SMB)	18 (2.5)
Age, y		Cytogenetics (CG)	28 (3.9)
18-29	103 (14.3)	Microbiology (M/SM)	14 (1.9)
30-39	244 (34.0)	Surgical Pathology/Histology (PA/HTL/HT)	33 (4.6)
40-49	184 (25.6)	Blood Banking/Chemistry/Hematology	28 (3.9)
50-59	105 (14.6)	Pathologist/Professor/ Researcher	45 (6.3)
60+	74 (10.3)	Other (including multiple disciplines)	24 (3.3)
Prefer not to answer	8 (1.1)	Laboratory role	
Ethnicity		Laboratory Assistant	23 (3.2)
Caucasian (White)	545 (75.9)	Technician/Technologist	315 (43.9)
African-American	39 (5.4)	Technician/Technologist—Lead	122 (17.0)
Latino or Hispanic	26 (3.6)	Supervisor/Manager	149 (20.8)
Asian	43 (6.0)	Laboratory Director/Administrator	41 (5.7)
Native American	3 (0.4)	Pathologist	35 (4.9)
Native Hawaiian/Pacific Islander	2 (0.3)	Other (PA, LIS, Quality, Education, etc)	33 (4.6)
Other/unknown/prefer not to say	25 (3.5)	Experience, y	
Multiple reported ethnicities	35 (4.9)	<1	16 (2.2)
Employment		1-3	80 (11.1)
Full-time	628 (87.5)	4-9	180 (25.1)
Part-time	40 (5.6)	10-19	243 (33.8)
Per diem	34 (4.7)	20+	199 (27.7)
Retired	16 (2.2)	Laboratory type	
Education		Independent private or reference	71 (9.9)
High school diploma	16 (2.2)	Hospital-based	553 (77.0)
Associate's degree	76 (10.6)	Clinical outpatient	37 (5.2)
Bachelor's degree	405 (56.4)	Research/Public health	17 (2.4)
Master's degree	154 (21.4)	Academic facility	38 (5.3)
Doctoral degree	63 (8.8)		
Prefer not to say	4 (0.6)		

## Descriptive Results

### Multivariate Analysis of Variance 

There was homogeneity of variance-covariance matrices as assessed by Box’s test of equality of covariance matrices (*P* > .001) and homogeneity of variance as assessed by Levene’s test of homogeneity of variance (*P* > .05). Due to unequal sample sizes between categorical levels, Pillai’s trace multivariate statistical findings are presented.^23^ The difference between gender identity on the combined change readiness scores was statistically significant, *F*(9, 2142) = 4.107, *P* < .001; Pillai’s V = .051; partial η^2^ = .021; and Tukey post hoc tests showed that for ACC, females had higher mean scores than males (*P* < .001), whereas males had higher NCC mean scores than females (*P* = .003). The differences between highest level of education on change readiness were also statistically significant, *F*(15, 2136) = 2.579, *P* < .001; Pillai’s V = .053; partial η^2^ = .021; and participants who earned a bachelor’s or master’s degree had a higher mean score for ACC than those with an associate’s degree (*P* = .007 and *P* < .001, respectively). No statistically significant difference was identified between level of education and CCC or NCC mean scores. Differences between laboratory role on change readiness were significant, *F*(18, 2133) = 3.024, *P* < .001; Pillai’s V = .075; partial η^2^ = .031; and both laboratory director/administrator and supervisor/manager had significantly higher mean scores for ACC than laboratory assistants (*P* = .012 and *P* = .004, respectively). Supervisor/manager roles also had significantly higher mean ACC scores than technicians/technologists (*P* < .01). Technician/technologists had higher mean CCC scores than supervisor/managers and laboratory directors/administrators (*P* < .001 and *P* = .043, respectively). No significant differences were identified between laboratory role and mean NCC scores.

### Results for H1: Correlation

H1 states that there is a significant association between leadership style (defined by the MLQ) and change readiness (measured by the TCM of CTC/Employee Commitment Survey).

H1_0_ states that there is not a significant association between leadership style (defined by the MLQ) and change readiness (measured by the TCM) of CTC/Employee Commitment Survey).

The Cronbach’s alpha coefficient measured .903, indicating a high level of internal consistency for the combined MLQ-TCM instrument within this specific sample.^23^ Pearson product-moment correlations examined potential associations between overall leadership style scales, detailed leadership style subscales, leadership outcomes, and the 3 CTC scales (ACC, CCC, and NCC) (TABLE 2).

**TABLE 2.** Correlation: leadership style subscales by commitment to change scales (n = 718)

Variables		Transformational					Transactional		Passive-avoidant		Outcomes			Leadership style			Commitment to change		
		IIA	IIB	IM	IS	IC	CR	MBE-A	MBE-P	LF	EE	EFF	SAT	TL	Trans.	PAB	ACC	CCC	NCC
Idealized influence-attributed	Pearson correlation	1.000	.835^a^	.844^a^	.848^a^	.858^a^	.852^a^	.081^b^	−.735^a^	−.755^a^	.835^a^	.885^a^	.890^a^	.943^a^	.698^a^	−.781^a^	.116^a^	−.240^a^	.061
	Sig (2-tailed)		< .001	< .001	< .001	< .001	< .001	.029	< .001	< .001	< .001	< .001	< .001	< .001	< .001	< .001	.002	< .001	.100
Idealized influence-behavioral	Pearson correlation	.835^a^	1.000	.855^a^	.825^a^	.788^a^	.826^a^	.101^a^	−.681^a^	−.671^a^	.764^a^	.804^a^	.796^a^	.923^a^	.690^a^	−.708^a^	.114^a^	−.274^a^	.058
	Sig (2-tailed)	< .001		< .001	< .001	< .001	< .001	.007	< .001	< .001	< .001	< .001	< .001	< .001	< .001	< .001	.002	< .001	.123
Inspirational motivation	Pearson correlation	.844^a^	.855^a^	1.000	.830^a^	.803^a^	.832^a^	.031	−.704^a^	−.690^a^	.766^a^	.816^a^	.812^a^	.929^a^	.653^a^	−.731^a^	.095*	−.260^a^	.031
	Sig (2-tailed)	< .001	< .001		< .001	< .001	< .001	.406	< .001	< .001	< .001	< .001	< .001	< .001	< .001	< .001	.011	< .001	.409
Intellectual stimulation	Pearson correlation	.848^a^	.825^a^	.830^a^	1.000	.859^a^	.847^a^	.071	−.701^a^	−.704^a^	.792^a^	.840^a^	.833^a^	.936^a^	.689^a^	−.737^a^	.116^a^	−.272^a^	.044
	Sig (2-tailed)	< .001	< .001	< .001		< .001	< .001	.058	< .001	< .001	< .001	< .001	< .001	< .001	< .001	< .001	.002	< .001	.239
Individualized consideration	Pearson correlation	.858^a^	.788^a^	.803^a^	.859^a^	1.000	.849^a^	.068	−.669^a^	−.697^a^	.812^a^	.863^a^	.853^a^	.926^a^	.689^a^	−.716^a^	.101^a^	−.267^a^	.005
	Sig (2-tailed)	< .001	< .001	< .001	< .001		< .001	.067	< .001	< .001	< .001	< .001	< .001	< .001	< .001	< .001	.007	< .001	.889
Contingent reward	Pearson correlation	.852^a^	.826^a^	.832^a^	.847^a^	.849^a^	1.000	.063	−.693^a^	−.715^a^	.790^a^	.860^a^	.846^a^	.903^a^	.801^a^	−.738^a^	.107^a^	−.297^a^	−.005
	Sig (2-tailed)	< .001	< .001	< .001	< .001	< .001		.090	< .001	< .001	< .001	< .001	< .001	< .001	< .001	< .001	.004	< .001	.890
Management by exception-active	Pearson correlation	.081^b^	.101^a^	.031	.071	.068	.063	1.000	−.002	−.069	.050	.049	.020	.076^b^	.649^a^	−.037	−.014	.143^a^	.083^b^
	Sig (2-tailed)	.029	.007	.406	.058	.067	.090		.952	.064	.177	.187	.598	.043	< .001	.323	.700	< .001	.027
Management by exception-passive	Pearson correlation	−.735^a^	−.681^a^	−.704^a^	−.701^a^	−.669^a^	−.693^a^	−.002	1.000	.820^a^	−.670^a^	−.766^a^	−.756^a^	−.749^a^	−.530^a^	.955^a^	−.078^b^	.242^a^	.002
	Sig (2-tailed)	< .001	< .001	< .001	< .001	< .001	< .001	.952		< .001	< .001	< .001	< .001	< .001	< .001	< .001	.037	< .001	.967
Laissez-faire leadership	Pearson correlation	−.755^a^	−.671^a^	−.690^a^	−.704^a^	−.697^a^	−.715^a^	−.069	.820^a^	1.000	−.682^a^	−.795^a^	−.790^a^	−.756^a^	−.587^a^	.953^a^	−.068	.230^a^	.018
	Sig (2-tailed)	< .001	< .001	< .001	< .001	< .001	< .001	.064	< .001		< .001	< .001	< .001	< .001	< .001	< .001	.069	< .001	.628
Extra effort	Pearson correlation	.835^a^	.764^a^	.766^a^	.792^a^	.812^a^	.790^a^	.050	−.670^a^	−.682^a^	1.000	.844^a^	.835^a^	.853^a^	.633^a^	−.709^a^	.085^b^	−.231^a^	.055
	Sig (2-tailed)	< .001	< .001	< .001	< .001	< .001	< .001	.177	< .001	< .001		< .001	< .001	< .001	< .001	< .001	.022	< .001	.141
Effectiveness	Pearson correlation	.885^a^	.804^a^	.816^a^	.840^a^	.863^a^	.860^a^	.049	−.766^a^	−.795^a^	.844^a^	1.000	.929^a^	.904^a^	.685^a^	−.818^a^	.075^b^	−.279^a^	.017
	Sig (2-tailed)	< .001	< .001	< .001	< .001	< .001	< .001	.187	< .001	< .001	< .001		< .001	< .001	< .001	< .001	.045	< .001	.657
Satisfaction	Pearson correlation	.890^a^	.796^a^	.812^a^	.833^a^	.853^a^	.846^a^	.020	−.756^a^	−.790^a^	.835^a^	.929^a^	1.000	.899^a^	.657^a^	−.810^a^	.050	−.258^a^	−.011
	Sig (2-tailed)	< .001	< .001	< .001	< .001	< .001	< .001	.598	< .001	< .001	< .001	< .001		< .001	< .001	< .001	.182	< .001	.762
Transformational leadership	Pearson correlation	.943^a^	.923^a^	.929^a^	.936^a^	.926^a^	.903^a^	.076^b^	−.749^a^	−.756^a^	.853^a^	.904^a^	.899^a^	1.000	.734^a^	−.789^a^	.117^a^	−.282^a^	.043
	Sig (2-tailed)	< .001	< .001	< .001	< .001	< .001	< .001	.043	< .001	< .001	< .001	< .001	< .001		< .001	< .001	.002	< .001	.254
Transactional leadership	Pearson correlation	.698^a^	.690^a^	.653^a^	.689^a^	.689^a^	.801^a^	.649^a^	−.530^a^	−.587^a^	.633^a^	.685^a^	.657^a^	.734^a^	1.000	−.585^a^	.073^b^	−.141^a^	.046
	Sig (2-tailed)	< .001	< .001	< .001	< .001	< .001	< .001	< .001	< .001	< .001	< .001	< .001	< .001	< .001		< .001	.050	< .001	.222
Passive-avoidant behavior	Pearson correlation	−.781^a^	−.708^a^	−.731^a^	−.737^a^	−.716^a^	−.738^a^	−.037	.955^a^	.953^a^	−.709^a^	−.818^a^	−.810^a^	−.789^a^	−.585^a^	1.000	−.076^b^	.247^a^	.010
	Sig (2-tailed)	< .001	< .001	< .001	< .001	< .001	< .001	.323	< .001	< .001	< .001	< .001	< .001	< .001	< .001		.041	< .001	.786
Affective commitment to change	Pearson correlation	.116^a^	.114^a^	.095^b^	.116^a^	.101^a^	.107^a^	−.014	−.078^b^	−.068	.085^b^	.075^b^	.050	.117^a^	.073^b^	−.076^b^	1.000	−.329^a^	.309^a^
	Sig (2-tailed)	.002	.002	.011	.002	.007	.004	.700	.037	.069	.022	.045	.182	.002	.050	.041		< .001	< .001
Continuance commitment to change	Pearson correlation	−.240^a^	−.274^a^	−.260^a^	−.272^a^	−.267^a^	−.297^a^	.143^a^	.242^a^	.230^a^	−.231^a^	−.279^a^	−.258^a^	−.282^a^	−.141^a^	.247^a^	−.329^a^	1.000	.153^a^
	Sig (2-tailed)	< .001	< .001	< .001	< .001	< .001	< .001	< .001	< .001	< .001	< .001	< .001	< .001	< .001	< .001	< .001	< .001		< .001
Normative commitment to change	Pearson correlation	.061	.058	.031	.044	.005	−.005	.083^b^	.002	.018	.055	.017	−.011	.043	.046	.010	.309^a^	.153^a^	1.000
	Sig (2-tailed)	.100	.123	.409	.239	.889	.890	.027	.967	.628	.141	.657	.762	.254	.222	.786	< .001	< .001	

ACC, affective commitment to change; CTC, commitment to change; CCC, continuance commitment to change; CR, contingent reward; EE, extra effort; EFF, efficiency; IC, individualized consideration; II, idealized influence; IIA, II-attributed; IIB, II-behavior; IM, inspirational motivation; IS, intellectual stimulation; LF, laissez-faire leadership; MBE, management by exception; MBE-A, MBE-active; MBE-P, MBE-passive; SAT, satisfaction; TL, transformational leadership.

^a^Correlation is significant at the .01 level (2-tailed).

^b^Correlation is significant at the .05 level (2-tailed).

### Leadership Style Subscale by Leadership Style Scale Correlation

MLQ scale intercorrelations demonstrate significant strong correlations both within subscales and across broad scales. IIA is not only significantly and strong positively correlated with all other TL subscales; that is, IIA and IC (*r*[716] = .88, *P* < .001), but with the overall TL scale as well (*r[*716] = .94, *P* < .001). The TL subscales have moderate to strong positive correlations with overall transactional leadership. For example, IIA is strongly and positively correlated with transactional leadership, *r*(716) = .70, *P* < .001. All transformational and transactional leadership subscales, except MBE-A, have significant strongly negative correlations with PAB. An example of this case is IIA and PAB, *r*(716) = −.75, *P* < .001. Despite CR falling under the category of transactional leadership, the association is stronger under TL than transactional leadership, *r*(716) = .90, *P* < .001 and *r*(716) = .80, *P* < .001, respectively. Both subscales of PAB have significant and strongly positive correlations with PAB and strong negative associations with TL. For example, MBE-P and PAB (*r*[716] = .96, *P* < .001) and LF and TL (*r*[716] = -.76, *P* < .001). Both subscales are moderately and negatively associated with transactional leadership.

### Leadership Style by Outcomes Correlation

The overall TL average and transformational subscales are strongly and positively associated with the 3 leadership outcomes of EE, EFF, and SAT. All TL subscales are strongly and positively associated with the 3 leadership outcomes. For example, IIA is strongly and positively correlated with all leadership outcomes: EE, *r*(716) = .84, *P* < .001; EFF, *r*(716) = .89, *P* < .001; and SAT, *r*(716) = .89, *P* < .001. The overall transactional leadership average and CR subscale are strongly and positively associated with the 3 leadership outcomes; however, no statistically significant correlation was identified between MBE-A and leadership outcomes. Both PAB and its 2 subscales, MBE-P and LF, are moderate-strongly and negatively correlated with the 3 leadership outcomes.

### Leadership Style by Change Readiness Correlation

A significant weak positive correlation was identified between TL and ACC, *r*(716) = .12, *P* = .002; and a significant moderate negative correlation was identified between TL and CCC, *r*(716) = −.28, *P* < .001. All TL subscales (IIA, IIB, IM, IS, and IC) were also positively associated with ACC and negatively associated with CCC. A significant yet minimal positive correlation was identified between transactional leadership and ACC, *r*(716) = .07, *P* = .050; and a significant weak negative correlation was identified between transactional leadership and CCC, *r*(716) = −.14, *P* < .001. Significant correlations were identified for both transactional leadership subscales CR and MBE-A. A significant weak positive correlation was identified between CR and ACC, *r*(716) = .11, *P* = .004, and a significant moderate negative correlation was identified between CR and CCC, *r*(716) = −.30, *P* < .001. MBE-A differed from the remainder of the leadership subscales in that a significant albeit weak positive correlation was identified in NCC, *r*(716) = .08, *P* = .027. Between MBE-A and CCC, a significant weak positive correlation was identified, *r*(716) = .14, *P* < .001. No significant correlation was identified between MBE-A and ACC.

A significant yet minimal negative correlation was identified between PAB and ACC, *r*(716) = −.08, *P* = .041, and a significant moderate correlation was identified between PAB and CCC, *r*(716) = .25, *P* < .001, with similar patterns in PAB’s subscales. Statistically significant correlations were identified between leadership style and both ACC and CCC; however, no statistically significant correlations were identified between leadership style and NCC. Therefore, there is a significant association between leadership style and change readiness, and we reject the null hypothesis. Leadership subscale and CTC correlations generally follow the same pattern as overall leadership scale and CTC results. This further supports the significant association identified between leadership style and change readiness.

### Results for H2: Regression

The H2 states that leadership style (defined by the MLQ) is a significant predictor of change readiness (measured by the TCM of CTC/Employee Commitment Survey).

H2_0_ states that leadership style (defined by the MLQ) is not a significant predictor of change readiness (measured by measured by the TCM of CTC/Employee Commitment Survey).

**Figure 4.**
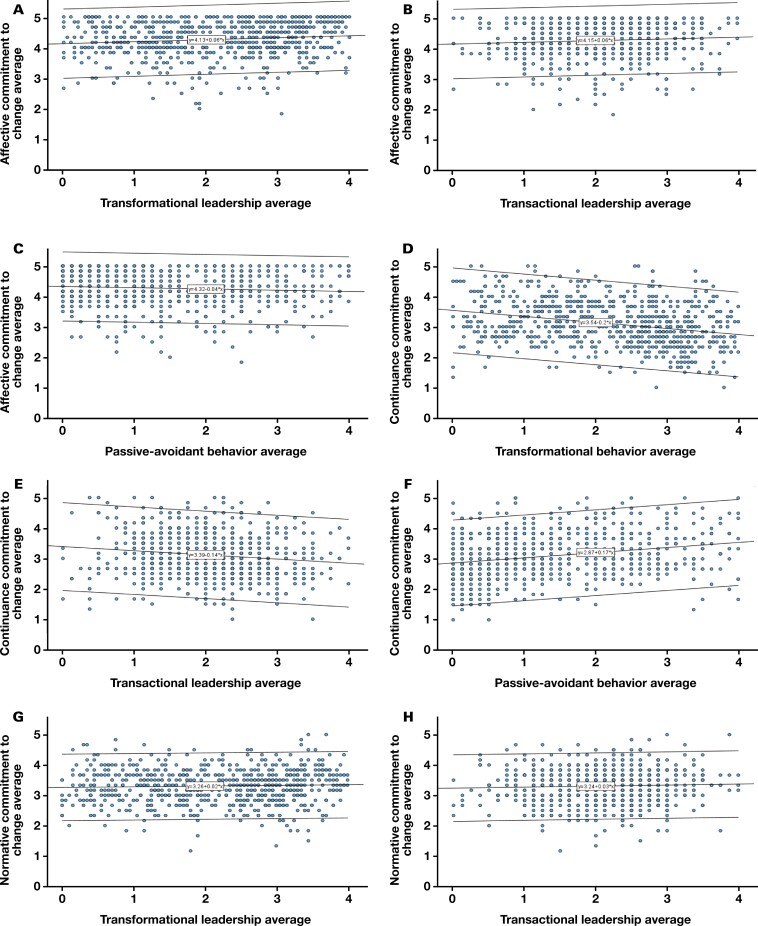

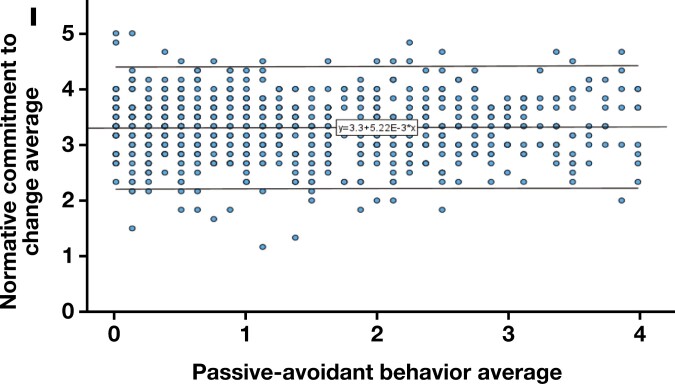
Multiple regression scatterplots. A, Affective commitment to change average by transformational leadership average (*R*^2^ = 0.014). B, Affective commitment to change average by transactional leadership average (*R*^2^ = 0.005). C, Affective commitment to change average by passive-avoidant behavior average (*R*^2^ = 0.006). D, Continuance commitment to change average by transformational leadership average (*R*^2^ = 0.079). E, Continuance commitment to change average by transactional leadership average (*R*^2^ = 0.020). F, Continuance commitment to change average by passive-avoidant behavior average (*R*^2^ = 0.061). G, Normative commitment to change average by transformational leadership average (*R*^2^ = 0.002). H, Normative commitment to change average by transactional leadership average (*R*^2^ = 0.002). I, Normative commitment to change average by passive-avoidant behavior average (*R*^2^ = 1.032E-4).

Three multiple regressions were performed to predict change readiness via ACC, CCC, and NCC from transformational, transactional, and passive-avoidant leadership styles. There was independence of residuals as assessed by a Durbin-Watson statistic of 1.936, 1.990, and 1.955, respectively. To determine linearity, scatterplots of CTC scales against leadership styles with a superimposed regression line were plotted (FIGURE 4).

There was homoscedasticity as assessed by visual inspection of a plot of standardized residuals vs standardized predicted values. Residuals were normally distributed as assessed by visual inspection of a normal probability plot; however, partial regression plots of NCC and leadership styles with superimposed regression lines did not exhibit linearity. Seven participants were outliers on the ACC scale with an average range of 1.83 to 2.50, 1 outlier with a CCC scale average of 1.33, and 4 outliers on the NCC scale with an average range of 1.17 and 5.00. They were not removed from the analysis despite the possibility of not representing the target population. Regression coefficients and standard errors can be found in TABLE 3.

**TABLE 3.** Multiple regression analysis

Change readiness		95% CI for *B*		SE B	β	*P* value	*R* ^*2*^	*∆R* ^*2*^
	B	LL	UL					
Affective commitment to change								
Model							.02	.01
Constant	4.074	3.84	4.31	.12		< .001		
Transformational	.093^a^	.01	.17	.04	.17	.020		
Transactional	−.020	−.10	.06	.04	−.03	.634		
Passive-avoidant	.022	−.04	.09	.03	.04	.500		
Continuance commitment to change								
Model							.09	.09
Constant	3.261	2.97	3.55	.15		< .001		
Transformational	−.235^b^	−.33	−.14	.05	−.33	< .001		
Transactional	.141^b^	.01	.24	.05	.14	.006		
Passive-avoidant	.047	.03	.12	.04	.07	.237		
Normative commitment to change								
Model							.01	.003
Constant	3.043	2.81	3.27	.12		< .001		
Transformational	.059	−.02	.13	.04	.11	.127		
Transactional	.024	−.06	.10	.04	.03	.554		
Passive-avoidant	.060	.00	.12	.03	.12	.055		

B, unstandardized regression coefficient; LL, lower limit; Model, “Enter” method in SPSS Statistics; UL, upper limit.

^a^P < .05.

^b^P *<* .001.

The first prediction equation was ACC = 4.07 + (0.093 × TL score) − (0.020 × transactional leadership score) + (0.022 × PAB score). The multiple regression model statistically significantly predicted ACC, F(3, 714) = 3.51, *P* = .015, accounting for 2.0% of the variation in ACC with adjusted *R*^2^ = 0.01. Only TL added statistically significantly to the prediction (*P* < .05). For CCC, the prediction equation was: CCC = 3.26 – (0.235 × TL score) + (0.141 × transactional leadership score) + (0.047 × PAB score). The multiple regression model statistically significantly predicted CCC F(3, 714) = 23.72, *P* < .001, accounting for 9.1% of the variation in CCC with adjusted *R*^2^ = 0.09. Transformational and transactional leadership styles added statistically significantly to the prediction (*P* < .01). For NCC, the prediction equation was: NCC = 3.04 + (0.059 × TL score) + (0.024 × transactional leadership score) + (0.060 × PAB score). Leadership style did not statistically significantly predict change readiness via NCC as there is no linear relationship, F(3, 714) = 1.77, *P* = .151, accounting for 0.7% of the variation in NCC with adjusted *R*^2^ = 0.003, which is virtually no size effect according to Cohen.^24^ Despite no significant relationship between leadership style and NCC, there were significant relationships between leadership style and both ACC and CCC. Leadership style is therefore a significant predictor of change readiness, and we reject the null hypothesis.

## Discussion

Regarding MANOVA findings, gender identity, education level, and laboratory role had significant yet minimal differences between average reported change readiness scores. For ACC, females had higher mean scores than males, whereas males had higher NCC mean scores than females, suggesting that females might be more intrinsically and autonomously motivated to commit to change based on self-determination theory when they perceive the leader to be supportive.^25^ Gender-related differences associated with self-determination theory can also justify that males experienced more of a moral obligation or controlled motivation to commit to change than females.^25^

Participants who earned a bachelor’s or master’s degree had a higher mean score for ACC than those with an associate’s degree, indicating that pursuing a higher level of education might also be associated with intrinsic motivation. Higher levels of education promote autonomy-supportive teaching, which promotes intrinsic motivation in students.^26^ Similarly, there may be an association between higher levels of intrinsic motivation and climbing a career ladder. Regarding laboratory role and ACC, both laboratory director/administrator and supervisor/manager roles had significantly higher mean scores for ACC than laboratory assistants, and supervisor/manager roles also had significantly higher mean ACC scores than technician/technologist. Where technician/technologists had higher mean CCC scores than supervisor/managers and laboratory director/administrators, this suggests that technicians and technologists may perceive there is too much at risk (loss of benefits, income, etc) to not accept the impending change compared with higher-level management who may be more secure in their positions.^27,28^ Lack of significant differences between laboratory role and mean NCC scores may indicate that, regardless of laboratory role, there is an inherent moral obligation to commit to change in the laboratory due to the nature of the profession.^27^

### Discussion of H1: Correlation

The positive correlation between TL and ACC suggests that followers may have higher levels of intrinsic motivation under transformational leaders, regardless of their dominant subscale. The opposite is also true, that TL and its 5 subscales are negatively correlated with CCC. The negative correlation is higher between IIB and CCC than the other subscales, suggesting that followers who perceive their leaders to exhibit higher levels of IIB are less likely to commit out of fear or negative feelings of risk. Lack of significant correlation between TL with associated subscales and NCC suggests that regardless of perceived TL level, it was not associated with higher or lower NCC. Additionally, the strong positive correlations between TL and the 3 leadership outcomes of EE, EFF, and SAT suggest that leaders who use TL have improved motivational skills, effective organizational interactions, and work with others in a satisfactory manner. These findings support the premise that TL improves job satisfaction, interpersonal interactions, and follower inspiration.^12^

Of the transactional leadership subscales, it is likely that CR is positively correlated with ACC and negatively correlated with CCC because leaders who express this subscale often use situational leadership where they vacillate between transactional and TL when appropriate.^1,29^ CR, like TL, was strongly and positively associated with leadership outcomes, which is likely also related to the use of situational leadership. The positive correlation between MBE-A and CCC is likely due to overlapping characteristics in leaders who express both MBE-A and MBE-P, the latter of which is characterized as a PAB.^12^ This is also supported by the fact that MBE leadership behaviors are corrective in nature and are associated with lower motivation.^12,16,17^

PAB’s negative correlation with ACC and positive correlation with CCC indicate that followers who perceive their leaders to exhibit nonleadership behavior are not intrinsically motivated to commit to change and instead commit only because they feel that that the cost of abandoning the organization is not worth the risk of not acceding the change. PAB is moderate-strongly and negatively correlated with the 3 leadership outcomes, suggesting that leaders who use PAB are more likely to produce negative outcomes, fail to inspire extra effort, be viewed as effective, or deliver satisfactory results.

The lack of significant correlation between leadership style and NCC suggests that a follower’s moral obligation to commit to change is unrelated to leadership style in the laboratory. However, both transformational and transactional leadership are positively correlated with ACC and negatively correlated with CCC, and PAB follows the opposite pattern. Medical laboratory followers under leaders who use any of the TL style subscales or the transactional leadership subscale of CR are more likely to report higher levels of intrinsic motivation to commit to change. In contrast, followers under leaders who exhibit MBE-A and PAB are more likely to report that they commit to change as a result of perceived cost.

### Discussion of H2: Regression

The multiple regression model significantly predicted ACC with only TL serving as a statistically significant predictor. Therefore, use of TL can positively predict higher levels of ACC in followers while having no causal or predictive relationship with CCC or NCC. This finding supports the premise of TL theory, in which leaders use this style to promote inspiration, intrinsic motivation, leadership attachment, and value alignment in their followers.^1,12^ The multiple regression model also significantly predicted CCC. Both transformational and transactional leadership styles added to the prediction in that use of TL results in lower levels of CCC, higher levels of transactional leadership without TL predicts higher levels of CCC, and lower values in TL predicts higher levels of CCC. The absence of a linear relationship between leadership style and change readiness via NCC explains the lack of correlation and predictive relationship between the 2 variables. This is likely due to the moral obligation or sense of duty that laboratory professionals and pathologists feel as health care providers to commit and remain loyal to organizational changes that promote quality.^27^ Despite the absence of a relationship between leadership style and NCC, there were significant predictor relationships between leadership style and both ACC and CCC, which carry implications for practice.

### Study Limitations

A methodological limitation of the study is the survey length, according to Portney and Watkins.^22^ Although the average completion time for this research survey was less than 8 minutes, it is likely that the 74-item survey was too long, resulting in a 73% completion rate. Other than limiting the number of demographic questions, removal of any of the scale items for the MLQ or the TCM could invalidate the results of the intended measures. Another limitation of the study is recruitment procedures. Although the survey was sent via email to ASCP and ASC members, it is possible that society members have restricted their communication preferences to limit survey emails. Additionally, the survey could have been sent to other medical laboratory and pathology professional societies. This approach could have increased recruitment for laboratory disciplines that had lower response rates, such as flow cytometry and microbiology, compared with medical laboratory science. Regarding participant demographics, a limitation is the lack of racial, ethnic, and gender diversity within the study sample. The survey was designed to promote equity in eligibility, and recruitment procedures did not demonstrate bias toward any 1 characteristic; however, there was an overwhelming representation of respondents reporting a Caucasian (White) ethnicity and a limited representation of respondents who identified as nonbinary or transgender. Although this finding was unexpected, recruitment procedures should have addressed potential barriers to better understand diversity, equity, and inclusion-related gaps in leadership and change readiness.

It is possible that extraneous factors affected leadership and commitment ratings. Highly motivated followers may perceive their leaders as transformational and overinflate their leaders’ TL scores rather than taking the survey through a more objective lens. Conversely, followers who are unsatisfied with their overall job, organizational culture, or senior leadership beyond their immediate manager may describe higher PAB in their direct leader rather than attributing other factors influencing their perspective. Similar external factors may influence their reported CTC, and the participant’s perspective of change may serve as a limiting feature. For example, some followers may perceive a change as minor, such as offering a new test or implementing new equipment, or major, such as a shift change or a hospital acquisition or merger. A change in leadership might also affect the follower’s responses, especially if leadership styles differ, therefore influencing change readiness responses. Previous negative experiences of change may alter change readiness responses even if more recent experiences were positive. To reduce variation, the CTC survey prompt should have specified “experiences regarding change ... under your current leader.”

### Implications for Practice

Leaders in laboratory medicine should immediately reflect on their current leadership style and determine whether behaviors incite intended commitment effects in their followers. Based on the findings of this study, it is recommended that laboratory leaders use TL and contingent reward styles to improve ACC and either transformational or transactional leadership to reduce CCC in their followers. If TL is the dominant leadership style but ACC is low and CCC is high, consider increasing IIB to reduce levels of CCC. If use of transactional leadership is necessary to offer structured management, formal delegation, and reinforcement systems, this style is still appropriate and effective at reducing CCC.^12,15^ However, consider situational leadership as the primary style where, depending on the situation, followers have the opportunity to develop higher levels of ACC under TL.^1,12^ The use of situational leadership may also improve leadership outcomes in the laboratory, as both TL and transactional leadership, including the CR subscale, are strongly and positively correlated with leadership outcomes of EE, EFF, and SAT.

Although LF did not serve as a predictor of CCC, its strong positive correlation with CCC suggests that use of the leadership style must be avoided to reduce the negative impact of CCC on their employees. Senior leadership must address LF in their subordinate managers as early as possible to reduce its potential downstream effects. It is also the responsibility of the follower to provide the leader with feedback of their experiences both individually and as a team, thus communicating potential gaps between leadership style and change readiness. Just as the MLQ can be used as both a leader self-assessment and as a follower’s assessment of the leader, the combined survey instrument may be used as an annual feedback tool. This combined survey instrument can also be used as the laboratory or health care organization anticipates additional changes, such as enrolling in a continuous improvement program or pivoting to meet new regulations.

Using TL to promote ACC and reduce CCC can mitigate barriers associated with change readiness, therefore promoting commitment to quality initiatives that promote high-value, patient-centered care. Laboratory teams that are affectively and intrinsically committed to change are more likely to embrace improvements within the laboratory and their organization that produce quality results for the patients.^30-32^ These improvements include engaged and accountable employees and increased efficiency by reducing turnaround time and errors or process variations in the total testing process.^33,34^ Consequently, clinicians and patients alike will benefit from improved accuracy and timeliness result delivery, leading to faster clinical decision-making and ideally, better patient outcomes.^12,34-36^

As health care organizations have a synergistic effect, improvements in change readiness within the laboratory will likely benefit other departments or disciplines within the organization.^30,37^ Interdisciplinary collaboration is better facilitated with motivated teams who have streamlined processes, and the laboratory that intrinsically commits to quality sets a precedent for other departments to follow.^30,37^ Although different organizational departments and health care disciplines may have unique demands when it comes to leadership, it would be helpful to observe leaders in laboratory medicine who use TL or a combination of both transformational and transactional leadership to incite change readiness via ACC and reduce levels of CCC. Other departments or health care disciplines can trial TL styles to determine whether there is an immediate benefit in their follower’s CTC.

### Future Research

As the first laboratory medicine study to examine the variables of leadership style and change readiness using the combined MLQ-TCM instrument, this study effectively established both associations and predictive relationships; however, a few notable gaps remain, encouraging additional research. Although the study involved a robust sample, it may prove worthwhile to replicate or modify the study to capture more data on marginalized populations or disciplines that were not sufficiently represented within this study. As all health disciplines must effectively collaborate to provide high-value, patient-centric care, this study should also be replicated across other health disciplines to determine consistency of findings and tailor leadership styles to specific disciplines, if applicable.

Additionally, future research should explore mediating factors between leadership style and change readiness. The association between laboratory passive behavior and CCC should be examined more closely. Nonleadership and CCC or total lack of commitment may have a snowball effect, negatively affecting other departments and, subsequently, patient care. Promoters of nonleadership and barriers of effectively using transformational, transactional, or situational leadership should be examined to determine whether internal factors within the leader or external restraints, such as insufficient resources in the laboratory, mediate this association. Because of the sensitive nature of NCC, with higher levels of NCC correlated with increased stress and emotional exhaustion, it is imperative to identify both internal and external factors outside of leadership style that may contribute to increased NCC and subsequent burnout in laboratory medicine.^10^ Most importantly, this study was completed during and within the early aftermath of the COVID-19 pandemic, and researchers must explore the long-term effects of the pandemic on leadership style and change readiness via CTC. It is recommended that the study be repeated within the next decade to explore differences in pandemic and postpandemic results within the laboratory. It is also important to understand how the COVID-19 pandemic affected leadership and CTC across multiple fields, including other health care disciplines and non–health care sectors.

## Conclusions

This quantitative study determined that leadership style is correlated with both change readiness via CTC and leadership outcomes in laboratory medicine. All subscales of TL along with the CR subscale of transactional leadership are positively correlated with ACC and negatively correlated with CCC. The reverse is also true, with PAB being negatively correlated with ACC and positively correlated with CCC. TL and transactional leadership are also strongly and positively correlated with the leadership outcomes of EE, EFF, and SAT, whereas PAB is strongly and negatively correlated with these outcomes. This study also determined that leadership style is a predictor of ACC and CCC but not NCC, and leaders should use TL to improve ACC and either TL or situational leadership to reduce CCC. Although the use of situational leadership can reduce CCC, it is likely that this leadership style will also increase follower’s ACC given that the situation calls for TL behavior. To inspire followers to embrace a change culture and be intrinsically motivated to support the continuous improvement mindset, it is recommended that leaders effectively use TL and contingent reward leadership. PAB serves as a barrier to intrinsic CTC and is associated with an intensified follower commitment out of stress or fear, further contributing to the field’s ever-increasing level of laboratory professional burnout and resistance to change. For the growth and well-being of the field and to remain competitive in the dynamic health care landscape, PAB should be limited or avoided, and TL along with contingent reward leadership should be embraced by leaders of laboratory medicine.

## Supplementary Material

lmad091_suppl_Supplementary_Material
