# Supplementary material for: Leading medical laboratory professionals toward change readiness: a correlational study
Source: Lab Med. 2023 Oct 3;55(3):255–66. doi: 10.1093/labmed/lmad091 (PMC11064098; doi:10.1093/labmed/lmad091)
Supplement: lmad091_suppl_Supplementary_Material [file lmad091_suppl_supplementary_material.docx]

Supplement

Laboratory Leadership & Readiness Survey

Consent

**1. Do you consent to participate in this research study?**A. Yes
B. No (ineligible for study)

Pre-Survey Eligibility Questions

**1. Do you work within the United States including its territories?**
A. Yes
B. No (ineligible for study)

**2. What is your current or most recent employment status?**
A. Full-Time
B. Part-Time
C. Per Diem
D. Retired
E. Student (ineligible for study)

**3. Are you proficient in reading the English language?**
A. Yes
B. No (ineligible for study)

**4. What is your age?**
A. Under 18 (ineligible for study)
B. 19-29
C. 30-39
D. 40-49
E. 50-59
F. Over 60
G. Prefer not to answer

Pre-Survey Demographic Questions

**1. What gender do you identify as?**
A. Female
B. Male
C. Transgender
D. Non-binary
E. Prefer not to answer
F. Other

**2. Please specify your ethnicity (check all that apply)**() Caucasian
() African-American
() Latino or Hispanic
() Asian
() Native American
() Native Hawaiian or Pacific Islander
() Other/Unknown
() Prefer not to say

**3. What is the highest degree or level of education you have completed?**
A. Some high school
B. High School Diploma or equivalent
C. Associate's Degree
D. Bachelor's Degree
E. Master's Degree
F. Doctorate Degree
G. Prefer not to say

**4. What is your laboratory discipline or ASCP certification (if applicable)?**
A. Cytology (CT/SCT)
B. Medical Laboratory Science (MLT/MLS)
C. Phlebotomy (PBT)
D. Molecular Biology (MB/SMB)
E. Flow Cytometry (SCYM)
F. Cytogenetics (CG)
G. Microbiology (M/SM)
H. Histology (HTL/HT)
I. Blood Banking/Chemistry/Hematology (BB/C/H/SBB/SC/SH)
J. Surgical Pathology (PA)
K. Pathologist/Professor/Researcher (MD/DO/MBBS/PhD/etc.)
L. Administration
M. Other (Specify):

**5. What is your role?**A. Laboratory Assistant
B. Technician/Technologist – Non-Supervisory
C. Technician/Technologist – Lead
D. Supervisor/Manager
E. Laboratory Director
F. Pathologist
G. Other (Specify):

**6. How many years of experience do you have as a laboratory professional?**
A. Less than 1 year
B. 1-3
C. 4-9
D. 10-19
E. 20+

**7. Where have you spent the majority of your career?**
A. Independent private or reference laboratory
B. Hospital-based laboratory
C. Clinical outpatient laboratory
D. Research laboratory
E. Public health laboratory
F. Academic facility – Laboratory professional program/medical school
G. Other (Specify)

Survey

Part I. For questions 1-45, think of your leader/manager or who you report to and use the following scale:

0: Not at all. 1: Once in a while. 2: Sometimes. 3. Fairly often. 4. Frequently, if not always.

THE PERSON I AM RATING. . .

1. Provides me with assistance in exchange for my efforts.................................................0 1 2 3 4

2. Re-examines critical assumptions to question whether they are appropriate .................0 1 2 3 4

3. Fails to interfere until problems become serious.............................................................0 1 2 3 4

4. Focuses attention on irregularities, mistakes, exceptions, & deviations from standards.0 1 2 3 4

5. Avoids getting involved when important issues arise .....................................................0 1 2 3 4

6. Talks about their most important values and beliefs.......................................................0 1 2 3 4

7. Is absent when needed.....................................................................................................0 1 2 3 4

8. Seeks differing perspectives when solving problems......................................................0 1 2 3 4

9. Talks optimistically about the future...............................................................................0 1 2 3 4

10. Instills pride in me for being associated with him/her ..................................................0 1 2 3 4

11. Discusses in specific terms who is responsible for achieving performance targets .....0 1 2 3 4

12. Waits for things to go wrong before taking action........................................................0 1 2 3 4

13. Talks enthusiastically about what needs to be accomplished........................................0 1 2 3 4

14. Specifies the importance of having a strong sense of purpose .....................................0 1 2 3 4

15. Spends time teaching and coaching ..............................................................................0 1 2 3 4

16. Makes clear what one can expect to receive when performance goals are achieved ...0 1 2 3 4

17. Shows that they are a firm believer in “If it ain’t broke, don’t fix it.” .........................0 1 2 3 4

18. Goes beyond self-interest for the good of the group .....................................................0 1 2 3 4

19. Treats me as an individual rather than just as a member of a group .............................0 1 2 3 4

20. Demonstrates that problems must become chronic before taking action .....................0 1 2 3 4

21. Acts in ways that builds my respect...............................................................................0 1 2 3 4

22. Concentrates their full attention on dealing with mistakes, complaints, and failures....0 1 2 3 4

23. Considers the moral and ethical consequences of decisions..........................................0 1 2 3 4

24. Keeps track of all mistakes ...........................................................................................0 1 2 3 4

25. Displays a sense of power and confidence....................................................................0 1 2 3 4

26. Articulates a compelling vision of the future ................................................................0 1 2 3 4

27. Directs my attention toward failures to meet standards ................................................0 1 2 3 4

28. Avoids making decisions...............................................................................................0 1 2 3 4

29. Considers me as having different needs, abilities, and aspirations from others............0 1 2 3 4

30. Gets me to look at problems from many different angles .............................................0 1 2 3 4

31. Helps me to develop my strengths ................................................................................0 1 2 3 4

32. Suggests new ways of looking at how to complete assignments ..................................0 1 2 3 4

33. Delays responding to urgent questions..........................................................................0 1 2 3 4

34. Emphasizes the importance of having a collective sense of mission ............................0 1 2 3 4

35. Expresses satisfaction when I meet expectations...........................................................0 1 2 3 4

36. Expresses confidence that goals will be achieved.........................................................0 1 2 3 4

37. Is effective in meeting my job-related needs.................................................................0 1 2 3 4

38. Uses methods of leadership that are satisfying .............................................................0 1 2 3 4

39. Gets me to do more than I expected to do.....................................................................0 1 2 3 4

40. Is effective in representing me to higher authority........................................................0 1 2 3 4

41. Works with me in a satisfactory way ............................................................................0 1 2 3 4

42. Heightens my desire to succeed ....................................................................................0 1 2 3 4

43. Is effective in meeting organizational requirements .....................................................0 1 2 3 4

44. Increases my willingness to try harder...........................................................................0 1 2 3 4

45. Leads a group that is effective ......................................................................................0 1 2 3 4


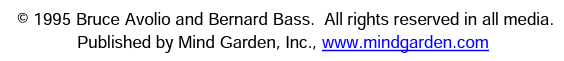


Part II. For questions 1-18, think of your experiences regarding changes within the laboratory or your organization. Use the following scale:

1. Strongly disagree. 2. Disagree. 3. Undecided. 4. Agree. 5. Strongly agree.

1. I believe in the value of change in the laboratory............................................................1 2 3 4 5

2. I feel a sense of duty to work toward change in the laboratory...................................... 1 2 3 4 5

3. I think that management is making a mistake by introducing change.............................1 2 3 4 5

4. Resisting change is not a viable option for me............................................................... 1 2 3 4 5

5. Things would be better without change.......................................................................... 1 2 3 4 5

6. Change is not necessary...................................................................................................1 2 3 4 5

7. I have no choice but to go along with proposed changes................................................1 2 3 4 5

8. I have too much at stake to resist change........................................................................ 1 2 3 4 5

9. Change serves an important purpose...............................................................................1 2 3 4 5

10. It would be irresponsible of me to resist change............................................................1 2 3 4 5

11. I do not think it would be right of me to oppose proposed changes..............................1 2 3 4 5

12. It would be risky to speak out against proposed changes..............................................1 2 3 4 5

13. I feel pressure to go along with proposed changes........................................................1 2 3 4 5

14. I would not feel badly about opposing change............................................................. 1 2 3 4 5

15. It would be too costly for me to resist change in my organization................................1 2 3 4 5

16. I would feel guilty about opposing this change.............................................................1 2 3 4 5

17. I do not feel any obligation to support this change........................................................1 2 3 4 5

18. Change is a good strategy for this organization. .......................................................... 1 2 3 4 5
